# Current ecology, not ancestral dispersal patterns, influences menopause symptom severity

**DOI:** 10.1002/ece3.5705

**Published:** 2019-11-05

**Authors:** Yuping Yang, Megan Arnot, Ruth Mace

**Affiliations:** ^1^ Life Sciences Lanzhou University Lanzhou China; ^2^ Department of Anthropology University College London London UK

**Keywords:** ecology, intragenomic conflict, kinship, menopause, menopause symptoms, residence patterns

## Abstract

All human females who reach midlife experience menopause, however, it is currently unclear why women experience this period of infertility, and why it is accompanied by many unpleasant symptoms. Using primary data from four ethnic groups in China, we test an existing theory that age of menopause and its symptoms are the result of intragenomic conflict between maternally and paternally inherited genes, with the outcome of such conflict predicted to be contingent on the ancestral postmarital residence pattern of the female (Úbeda, Ohtsuki, & Gardner, Ecology Letters, 17, 2014, 165). The model predicts that being ancestrally patrilocal results in less intragenomic conflict, causing a shorter, less symptomatic perimenopause that terminates in a later menopause. Our findings show no support for this hypothesis and suggest current, rather than ancestral, residence patterns better predict aspects of the menopausal transition. Furthermore, current patrilocality when compared to duolocality is associated with more severe menopause symptoms, which may be due to sexual, rather than intragenomic, conflict.

**Open Research Badges:**

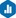

This article has earned an Open Data Badge for making publicly available the digitally‐shareable data necessary to reproduce the reported results. The data is available at https://doi.org/10.5061/dryad.27s8k0p.

## Graphical abstract

A previous model has suggested that intragenomic conflict is somewhat responsible for the symptoms experienced by women running up to the menopause, in addition to the timing of menopause itself. Using primary data from China, we find no support for this hypothesis, but do find that social and sexual conflict may play a role in peri‐menopause symptom severity.

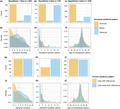

## 1 INTRODUCTION

Dispersal patterns structure the relatedness of individuals to the group, which can influence patterns of cooperation and conflict (Johnstone & Cant, 2010). Sex‐specific dispersal at marriage means that male and female relatedness to their residential group will vary with age, which means they may experience different evolutionary pressures over their lifespan. The human female life history is characterized by menopause, which is an extended period of infertility that usually occurs around the age of 50. While there is a great deal of variation in age of natural menopause (ANM) both within and between populations (Sievert, 2014), it is not uncommon for a woman to spend around a third of her life postmenopausal (Khaw, 1992; Laisk et al., 2019; Mace, 2000).

As the trait has apparent negative consequences on the woman's direct fitness, it has received a great deal of attention from evolutionary biologists and anthropologists who seek to understand why selection would have actively favoured prolonged postreproductive survival in women. Many agree that menopausal women may be able to increase their indirect fitness through consanguinial cooperative breeding (e.g., grandmothering) when there are age‐specific relatedness asymmetries between the women in the group (Cant & Johnstone, 2008; Hawkes, 2003, 2004; Hawkes, O'Connell, Blurton Jones, Alvarez, & Charnov, 1998; Johnstone & Cant, 2010; Kim, Coxworth, & Hawkes, 2012; Sear, Mace, & McGregor, 2000), and some have argued that dispersal patterns were crucial to setting up the reproductive conflict that facilitated the evolution of the menopause itself (Cant & Johnstone, 2008; Johnstone & Cant, 2010). Although, it is unclear whether the indirect fitness benefits of grandmothering under certain residence patterns are enough to select for an extended period of infertility (Lahdenperä, Mar, & Lummaa, 2016; Rogers, 1993; Shanley, Sear, Mace, & Kirkwood, 2007; Snopkowski, Moya, & Sear, 2014). Furthermore, it has recently been brought into question by Úbeda, Ohtsuki, and Gardner (2014) that if an extended postreproductive lifespan is an adaptive feature of the human female life history, then why is it not a smooth transition from fertility to infertility, and such an onerous process for many women?

While there is a great deal of variation in menopause symptom experience, they are a ubiquitous complaint among menopausal women, with common symptoms including hot flashes, vaginal dryness, joint and muscle pain, and anxiety (Avis, Stellato, et al., 2001; Islam, Bell, Rizvi, & Davis, 2017). Mechanistically, menopause symptoms can be understood to be largely the result of hormonal fluctuations (Douma, Husband, O'Donnell, Barwin, & Woodend, 2005; Prior, 1998, 2006), but there is little understanding of whether there is any adaptive evolutionary rationale underlying the presentation of these symptoms.

Recently, it was proposed that a woman's experience during the menopausal transition, and the age at which she reaches physiological reproductive cessation, may be the result of intragenomic conflict (Úbeda et al., 2014). Intragenomic conflict refers to the evolutionary phenomenon in which selection favors genes with opposing expressions in the same individual (Gardner & Ubeda, 2017). In the case of menopause, it is modelled that genes related to fertility will have parent‐of‐origin differential expression, with paternally inherited genes favouring the earlier cessation of fertility, whereas maternally inherited genes would promote the converse (Úbeda et al., 2014). When there are high rates of female dispersal for reproduction at marriage (i.e., patrilocal societies), competition between maternally related kin is relaxed, hence maternally inherited genes promoting fertility (e.g., those that slow follicular atresia) are expressed, resulting in the woman experiencing a later menopause. However, when females do not disperse for reproduction (i.e., live under a duolocal or matrilocal social structure), then maternal kin are in more competition with each other and their maternally inherited genes are silenced resulting in an earlier menopause. Furthermore, the model states that under low rates of female dispersal, paternally inherited genes are expressed stochastically because the optimal age at menopause is more divergent between maternal and paternal genes in matrilocal systems, to which Úbeda et al. (2014) attribute the negative symptoms that accompany the perimenopause. Hence, the model of intragenomic conflict applied to the menopausal transition predicts that women from populations that ancestrally displayed high rates of female dispersal should experience:

1. Less severe symptoms in the period approaching menopause (known as the perimenopause);
2. A shorter peri‐menopause; and
3. A later menopause.

This model has not been directly tested. Indirect evidence comes from research demonstrating that ethnicity is associated with menopause symptoms severity and timing. Women of Japanese descent in the USA typically report less vasomotor symptoms (e.g., hot flashes) and a later ANM, whereas African American women experience the converse (Avis, Stellato, et al., 2001; Avis et al., 2005; Gold et al., 2001, 2000; Im, 2009). However, both groups would have experienced a degree of admixture in their past, and as noted by Úbeda et al. (2014), good evidence on the ancestral ecologies of these populations is lacking. Some ethnographic reports state that Japan was traditionally patrilineal and patrilocal (Befu, 1968), offering some support for the hypothesis. Although, the ancestry of African Americans is diverse, and there are conflicting reports on the ancestral residence pattern of various African groups (e.g., opposing findings on Bantu residency see: Hage & Marck, 2015; Opie, Shultz, Atkinson, Currie, & Mace, 2014). Additionally, when testing an alternative theory of the evolution of menopause, Snopkowski et al. (2014) presented results that found no evidence that ancestrally patrilocal women have an earlier ANM in Indonesia.

To formally test the intragenomic conflict hypothesis, we collected data from southwestern China. This area includes diverse populations where there is variation in kinship systems, which determine the degree of female dispersal at marriage (Wu, Ji, He, Du, & Mace, 2015). In most Chinese kinship systems, males never disperse, but there is some variation in female dispersal: in patrilineal groups females disperse at marriage, whereas in matrilineal groups they usually stay in their natal households and live under a duolocal residence pattern, where neither males nor females disperse. However, there is always variability in residence patterns around the norm (Ly et al., 2018), and many women who are ancestrally duolocal no longer live in this way due to cultural change in the region (Ji et al., 2016; Mattison, 2010); therefore, we collected data on current residence pattern as well. This second variable allows us to attempt to capture whether the ancestral or current residence pattern is more influential over the menopausal transition. The intragenomic conflict hypothesis predicts that the former would be most important, whereas if conditions specific to current residence pattern (e.g., different degrees of sexual and social conflict between patrilocal and duolocal groups (Leonetti, Nath, & Hemam, 2007)) are more important, then menopause symptoms may be more related to current living arrangements.

## 2 MATERIAL AND METHODS

### 2.1 Study area

Data collection was carried out over two field sessions in the Sichuan Province of China in 2018 and 2019. Consent was sought from each individual interviewed and from local People's Government at each site, and the research was approved by Lanzhou University Life Sciences and UCL Research Ethics committee. In each site, different ethnic groups were interviewed, with Mosuo, Han and Yi women being interviewed around Lugu Lake and Zhaba women in Daofu County. These different ethnic groups have likely been living this way for thousands of years (Dong, Yu, & Liu, 2008) and therefore serve as good models for testing the intragenomic conflict hypothesis. The Mosuo and Zhaba women display an uncommon duolocal residence pattern with matrilineal descent, where neither sex leaves the natal group for marriage, and descent is traced through the female line. As an alternative to cohabiting marriage, they engage in the practice of *zǒu hūn* (“walking marriage,” 走婚), in which males and females live in their natal households that comprise of many generations of family members (He, Wi, Ji, Tao, & Mace, 2016; Ji et al., 2013; Wu, Ji, et al., 2015), and men only visit their wives and girlfriends during the night (Cai, 2001). Men have little or no financial obligations to their spouses or children, and they tend to invest heavily in their sisters' offspring (He, Wu, Ji, Tao, & Mace, 2016; Ji et al., 2016). A family planning policy established in the 1980s meant that marriage became more important for reproduction, and therefore, partnerships in the Mosuo and Zhaba are slightly more formal now than they were in previous years; however, in most cases, the husband and wife still live apart (Ji et al., 2016; Thomas et al., 2018). As a contrast to these groups with little female dispersal, the Han and the Yi display patrilocality accompanied by patriliny where descent is traced down the male line, and women leave their natal home to join that of their husbands' kin at marriage.

As residence patterns were always somewhat flexible (Ly et al., 2018) and have become more fluid in recent years, both due to policy changes and increased tourism (Mattison, 2010), this enables us to consider the individuals ancestral residence pattern (based on their ethnic group) in addition to the way in which they are currently living. The intragenomic conflict hypothesis predicts that ancestral residence pattern should be influential over menopause timing and symptoms (Úbeda et al., 2014), however, if current residence pattern better predicts variation in menopause timing and symptoms, then it would suggest something else is responsible for the diversity we see in the menopausal transition.

### 2.2 Data collection

A standard demographic survey was conducted in eight villages around Lugu Lake and five villages in Daofu County. All houses in the area were visited, and in households that agreed to participate adult women were interviewed, with demographic data including data on living arrangements, marital status, financial security, fertility status, parity etc. being collected. We also collected data on aspects of the menopausal transition using the Menopause Rating Scale (MRS; see Figure S1; Heinemann, Potthoff, & Schneider, 2003), which was developed to ensure clinicians and researchers had a standardized method of looking at the quality of life of women passing through the menopausal transition. While the MRS was developed by Western clinicians, it has been cross‐culturally validated both in Western and non‐Western societies (Dinger, Zimmermann, Heinemann, & Stoehr, 2006; Heinemann et al., 2003; Wu, Wen, Hwang, & Huang, 2015). Furthermore, the understanding of the menopause within the four groups of interest is comparable to that of Western culture. In Chinese, the menopause can be translated as *juéjīng* (絕經), which refers to the cessation of menstruation, and the perimenopause is understood to be a gradual process and is known as as *gēngniánqí* (更年期). However, through using a questionnaire in which the categories of menopause symptoms are imposed upon the interviewees rather than emerging through unstructured interviews, it is slightly restrictive in that other symptoms that are not included within the MRS will be excluded, although it does allow us to compare it to existing literature on the perimenopause. The MRS measures a total of 11 symptoms, with each symptom being rated by the woman according to its severity using a likert scale ranging from 0 to 4 (none; mild; moderate; severe; and very severe). Symptoms can be divided into three subgroups: psychological symptoms (4 symptoms: depression, irritability, anxiety, and exhaustion), urogenital symptoms (3 symptoms: sexual problems, bladder problems, and vaginal dryness), and somato‐vegetative symptoms (4 symptoms: hot flushes, heart discomfort, sleep problems, and joint and muscular discomfort). Using the measures derived from the likert scale, an overall symptoms score can be calculated (ranging from 0 to 44), in addition to individual symptom scores (psychological: 0 to 16; urogenital: 0 to 12; somato‐vegetative: 0 to 16). Symptom reporting could either be current or retrospective, with women who had already passed through the transition being asked how severe their symptoms were when they were experiencing perimenopause, and women who are perimenopausal being asked about their current experience. This means when modelling symptom severity in peri and postmenopausal slightly different things will be captured: for postmenopausal women, it can be seen we are modelling an average of their overall menopausal experience, whereas for perimenopausal women it is a snapshot of their current symptoms.

### 2.3 Variables

#### 2.3.1 Variables of interest

In order to test the intragenomic conflict hypothesis of the menopausal transition, three variables of interest were collected: severity of perimenopause symptoms, the duration of the perimenopause symptoms, and the ANM. 

Firstly, when looking at the severity of symptoms, only data on somato‐vegetative symptoms were used. This is primarily because the hypothesis we are testing models the effect of residence on vasomotor symptoms, which include hot flashes and night sweats. The MRS measures somato‐vegetative symptoms, which includes hot flashes, but also groups them with heart discomfort, sleeping issues, and joint and muscle complaints; all of which have been found to be exacerbated by, and symptomatic of, vasomotor issues (Ashraf et al., 2015; Avis, Crawford, Stellato, & Longcope, 2001; Chung et al., 2018; Islam, Bell, Billah, Hossain, & Davis, 2016). Therefore, to adhere to the assumptions of the model, we limited the analysis to somato‐vegetative symptoms, in which we summed the responses to the relevant questions, giving us a symptoms severity score ranging from 0 to 16, where a higher number indicates worse symptoms.

Symptom duration was measured through asking women at what age or in what year they began experiencing menopause symptoms. If they had already entered menopause then their age at menopause was used as their symptom finishing date, and if they had not yet entered menopause then they were coded as ongoing. We acknowledge that menopause symptoms are not confined to the pre and perimenopause and can often persevere following the termination of fertility (Avis et al., 2015); however, the hypothesis of interest specifically models symptoms up to and until the final menstruation. Therefore, while some women did report that they were still experiencing symptoms following the cessation of fertility, the duration of their symptoms was only included until menopause.

Finally, ANM was self‐reported through asking women whether they were experiencing regular or irregular menstruation, and when they had experienced their last period. If a woman had not experienced a period for 12 months or more in the absence of extenuating factors (e.g., pregnancy, breastfeeding etc.), then she was considered to have experienced menopause (Kirchengast & Rühli, 2013). Furthermore, if a woman was reporting irregular periods then she was classed as being perimenopausal and anyone with regular periods or reproducing (i.e., pregnant) as premenopausal.

#### 2.3.2 Residence pattern

The intragenomic conflict hypothesis predicts that more female dispersal will result in a menopausal transition characterized by less severe symptoms that occur for a shorter period of time and terminate in a later menopause. In order to test this, we use two variables indicative of the individual's residence pattern. Firstly, their ancestral residence pattern, which was derived from their self‐reported ethnicity. Women who reported being Mosuo or Zhaba were listed as having a duolocal ancestral residence pattern, and those who identified as Han or Yi were ancestrally patrilocal. In addition, a small proportion of women reported mixed ancestry. This variable was included to capture the specific predictions of Úbeda et al. (2014)'s model, in which they modelled lower rates of female dispersal against higher rates. For example, if a woman reports being Han, then it can be inferred that her ancestral residence pattern was primarily female dispersing (i.e., high rates of female dispersal).

We also included a second variable of current residence pattern. This indicates how the woman lives currently, regardless of her ethnicity or ancestral residence pattern, and allows us to understand separate effects of current versus ancestral living conditions. Here, women were either coded as living with (i.e., duolocally), or away from (i.e., patrilocally), their natal household.

#### 2.3.3 Covariates

Covariates were held constant in each analyses and selected based on existing literature on the demographic correlates of menopause symptoms and timing, and also based on the data we had available. These included parity (Li et al., 2012; Mishra et al., 2017; Parazzini, 2007; Wang et al., 2018), whether the woman was financially secure over the past year (as a measure of socioeconomic position/financial stress; Lawlor, Ebrahim, & Smith, 2003; Schoenaker, Jackson, Rowlands, & Mishra, 2014), body mass index (BMI; Ahuja, 2016; Li et al., 2012; Maru, Verma, Verma, & Shrimal, 2016; Parazzini, 2007; Sapre & Thakur, 2014; Tao et al., 2015; Wang et al., 2018; Zhu et al., 2018), and smoking habits (Ayatollahi, Ghaem, & Ayatollahi, 2005; Bjelland, Hofvind, Byberg, & Eskild, 2018; Gold et al., 2001, 2000; McKnight et al., 2011; Nagel, Altenburg, Nieters, Boffetta, & Linseisen, 2005; Özdemir & Çöl, 2004; Parazzini, 2007; Sapre & Thakur, 2014; Tao et al., 2015; Wang et al., 2018). In addition, age was included in the model when looking at symptom duration and symptom severity, and menopause status when looking at symptom severity, to control for the possibility that retrospective accounts of menopause may differ to current reporting.

### 2.4 Analyses

Different datasets with different sample sizes were used for each analysis. This is in part due to data being limited as a result of fieldwork constraints, in addition to the fact that different samples of women are required to test each hypothesis. When looking at symptom severity, only women who were perimenopausal were included in the analysis; women who could not recall when they started experiencing menopause symptoms or had never experienced menopause symptoms were excluded when looking at symptom duration; and no women were removed when looking at ANM. As there are varying sample sizes which included different women, it means the results from each analysis are not necessarily directly comparable to one another. Furthermore, the different sizes of dataset means that each analysis has different levels of statistical power. With alpha set at 0.05 and power at 0.80, the datasets used to look at ANM (*n* = 876), symptom severity (*n* = 445), and symptom duration (*n* = 83) have enough power to capture small, medium, and large effect sizes, respectively (Cohen, 1992).

To test the first hypothesis that women from ancestrally patrilocal societies should experience less severe menopause symptoms, Poisson regression was used to account for the fact that the outcome variable is a count variable with a non‐normal distribution. To test the latter two hypotheses that ancestral patrilocality associates with a shorter perimenopause and a later menopause, Cox proportional hazards models were created to calculate a hazard ratio (HR) for the risk of the perimenopause ending and menopause occurring, respectively (Cox, 1972). To test the former hypothesis, symptom duration in years was used as the timescale within the model, with the participant being censored if she had not yet entered menopause and was still reporting symptoms and the event being coded as having occurred once the woman reached menopause. When looking at ANM, the age of the woman was used as the timescale (Korn, Graubard, & Midthune, 1997), with women who had not yet entered menopause (pre and perimenopausal) or ceased fertility for another reason (e.g., pregnancy) being censored.

When testing each hypothesis, univariate analysis was initially carried out, and then, three separate models tested. In the first model, both ancestral and current residence patterns were included (alongside the other covariates), with subsequent models containing firstly only current residence pattern and secondly only ancestral residence pattern. Models were then compared using the Akaike Information Criteron (AIC), in which the model with the lowest AIC value is the best fit for the data. Models were then weighted based on how much their AIC value increase compared to the model that best fit the data, in which a decrease in AIC value of two or more implies a better model fit (Burnham & Anderson, 2002). All analyses were carried out in *R* (version 3.5.3) using the packages *AICcmodavg*, *survival*, and *survminer* (Kassambara & Kosinski, 2018; Mazerolle, 2019; R Core Team, 2019; Therneau, 2015).

## 3 RESULTS

### 3.1 Hypothesis 1. More female dispersal associates with a less symptomatic perimenopause

**Figure 1:**
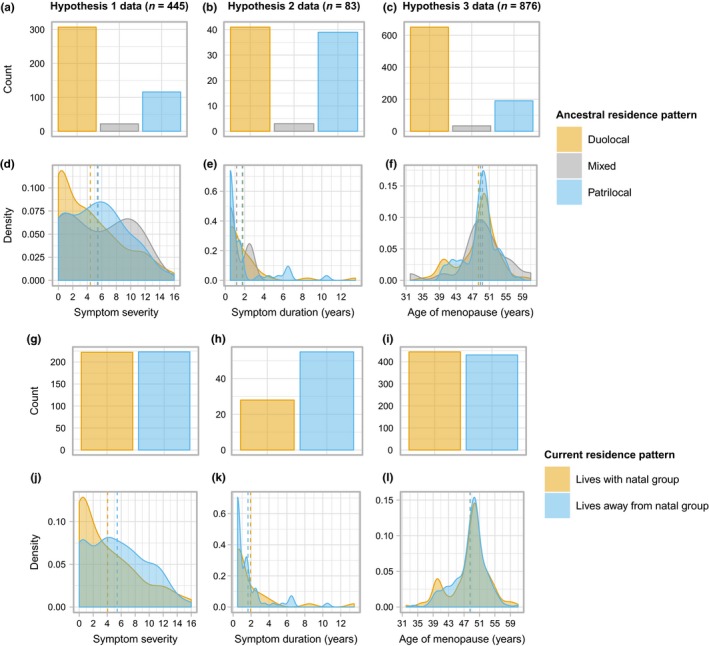
Descriptive statistics for each dataset. Plots a, d, g, and j show the amount of women from each ancestral (a) and current (g) residence pattern in the dataset used to look at symptom severity and the distribution of their somato‐vegetative symptom scores (d and j). Plots b, e, h, and k show the amount of women from each ancestral (b) and current (h) residence pattern and the distribution of their symptom duration by residence pattern (e and k). Plots c, f, i, and l show the amount of women from each ancestral (c) and current (i) residence pattern in the dataset used to look at menopause timing and the distribution of age of menopause by residence pattern (f and l). Dashed lines indicate mean value by group

To test whether patrilocality associates with a less symptomatic perimenopause, data from 445 women were used. In this sample, the average age was 60.49 (standard deviation [*SD*] = 9.06), and the women were primarily postmenopausal (90%), meaning that the majority of the symptom reporting was retrospective. The sample here was biased toward women who are ancestrally duolocal (69%; Figure 1a), who also presented a lower average symptoms score (4.41, *SD* = 4.33) than women from patrilocal (5.49, *SD* = 4.09) or mixed descent (5.41, *SD* = 4.52; Figure 1d). The majority of women in the sample had never smoked (93%); however, a larger proportion of ancestrally patrilocal women reported having ever smoked (16%) than women from duolocal (4%) or mixed ancestry (0%). Full descriptive statistics are presented in Table 1.

**Table 1:** Descriptive statistics for dataset used to test whether menopause symptom severity associates with ancestral patrilocality

	Total		Ancestral residence pattern					
			Duolocal (*n* = 307)		Mixed (*n* = 22)		Patrilocal (*n* = 116)	
	*n* (%)	Mean (*SD*)	*n* (%)	Mean (*SD*)	*n* (%)	Mean (*SD*)	*n* (%)	Mean (*SD*)
Somato‐vegetative symptom score		4.74 (4.30)		4.41 (4.33)		5.41 (4.52)		5.49 (4.09)
Age		60.49 (9.06)		59.85 (9.26)		63.32 (7.92)		61.66 (8.58)
Menopause status								
Perimenopausal	46 (10.3)		36 (11.7)		0 (0.0)		10 (8.6)	
Postmenopausal	399 (89.7)		271 (88.3)		22 (100.0)		106 (91.4)	
Current residence pattern								
Lives with natal group	222 (49.9)		201 (65.5)		16 (72.7)		5 (4.3)	
Lives away from natal group	223 (50.1)		106 (34.5)		6 (27.3)		111 (95.7)	
Ancestral residence pattern								
Duolocal	307 (69.0)		307 (100.0)		0 (0.0)		0 (0.0)	
Mixed	22 (4.9)		0 (0.0)		22 (100.0)		0 (0.0)	
Patrilocal	116 (26.1)		0 (0.0)		0 (0.0)		116 (100.0)	
Parity		3.86 (1.92)		3.62 (1.77)		4.68 (2.40)		4.34 (2.09)
Experienced financial difficulty in past year								
Yes	266 (59.8)		171 (55.7)		15 (68.2)		80 (69.0)	
No	179 (40.2)		136 (44.3)		7 (31.8)		36 (31.0)	
Ever smoked								
Yes	31 (7.0)		12 (3.9)		0 (0.0)		19 (16.4)	
No	414 (93.0)		295 (96.1)		22 (100.0)		97 (83.6)	
BMI		22.91 (4.79)		23.40 (5.17)		22.00 (3.54)		21.79 (3.59)

Abbreviations: BMI, body mass index; *SD*, standard deviation.

**Figure 2:**
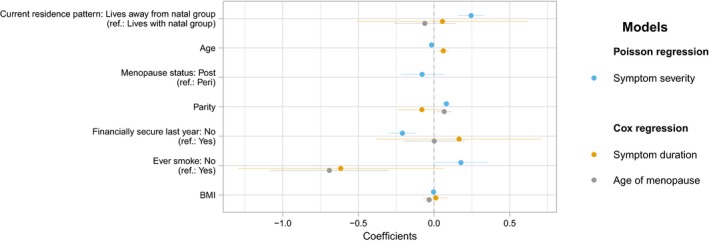
Dot and whisker plot showing the coefficients for each variable in the model. A higher coefficient indicates (1) more severe symptoms, (2) a shorter symptom duration, and (3) an earlier menopause. Note: Hazard ratios described in text and in Appendix S1 are the exponential of the coefficient

Univariate Poisson regression models indicate that patrilocality both currently (*β* = 0.29, standard error [*SE*] = 0.04) and ancestrally (*β* = 0.22, *SE* = 0.05) associates with worse menopause symptoms. However, following complete adjustment, ancestral residence pattern loses its significance within the model, but current residence pattern still has a relationship with symptom severity (*β* = 0.23, *SE* = 0.05). Model fitting (Table 2) suggests that Model 2 (Table 3), which did not include ancestral residence pattern, was the best fitting model, with results still indicating that women who lived dispersed from their natal group experience more severe menopause symptoms (*β* = 0.25, *SE* = 0.04). Results also suggested that symptoms were worse for women who have experienced financial insecurity and have more children. Full results are presented in the Table S1, and best model results displayed graphically in Figure 2.

**Table 2:** Results from model selection based on Akaike Information Criteron (AIC)

Hypothesis	Model	*K*	AIC	ΔAIC	*w_i_*
H1. Ancestral patrilocality results is less severe menopause symptoms	Current and ancestral residence pattern	10	3,064.07	0.10	0.49
	**Current residence pattern**	**8**	**3,063.97**	**0.00**	**0.51**
	Ancestral residence pattern	9	3,081.30	17.34	0.00
H2. Ancestral patrilocality results in a shorter symptom duration	Current and ancestral residence pattern	8	501.68	3.18	0.12
	**Current residence pattern**	**6**	**498.49**	**0.00**	**0.57**
	Ancestral residence pattern	7	499.68	1.19	0.31
H3. Ancestral patrilocality results in a later age of menopause	Current and ancestral residence pattern	7	4,157.16	61.84	0.00
	**Current residence pattern**	**6**	**4,095.32**	**0.00**	**0.73**
	Ancestral residence pattern	7	4,097.27	1.95	0.27

Note

All models adjust for parity, financial security, smoking status, and body mass index, with Hypothesis 1 (H1) and Hypothesis 2 (H2) models adjusting for age, and H1 models adjusting for menopausal status. Best fitting model (which has an AIC change of 0) for each hypothesis are highlighted in bold.

Abbreviations: H3, hypothesis 3; *K*, number of parameters; *w_i_*, model probability.

**Table 3:** Results from the best fitting model when testing the association between ancestral residence pattern and symptom severity

	*β* (*SE*)	*p*
Current residence pattern (ref.: Lives with natal group)		
Lives away from natal group	0.25 (0.04)	<.01
Age	−0.02 (0.00)	<.01
Menopause status (ref.: Peri)		
Postmenopausal	−0.08 (0.07)	.27
Parity	0.08 (0.01)	<.01
Experienced financial difficulty in past year (ref.: Yes)		
No	−0.21 (0.05)	<.01
Ever smoked (ref.: Yes)		
No	0.18 (0.09)	.05
BMI	0.00 (0.01)	.51

Note

Ancestral residence pattern was not retained in the best fitting mode, whereas current residence pattern was, with women who leave their natal group to marry experiencing more severe symptoms (*n* = 445).

Abbreviations: BMI, body mass index; *SE*, standard error.

### 3.2 Hypothesis 2. More female dispersal associates with a shorter perimenopause

To test hypothesis 2, data on a sample of 83 women were used, of which 49% were ancestrally duolocal (Figure 1b). The majority were postmenopausal (84%) with a mean age of 60.24 (*SD* = 7.17). On average, women experienced menopause symptoms for 1.75 years (*SD* = 2.32), with ancestrally duolocal women experiencing symptoms for slightly longer (1.80 years, *SD* = 2.42; Figure 1e). See Table S2 for full descriptive statistics.

Cox regression was employed here to measure the difference in symptom duration. Both prior to and after adjustment, there was no evidence that either ancestral or current residence pattern impacted menopause symptom duration (Table S3). However, when comparing models (Table 2), the model that included only current residence pattern was the best fit for the data (Figure 2). In all models, women who had never smoked experienced perimenopausal symptoms for a longer period of time (full model HR = 0.50, *p* = .05).

### 3.3 Hypothesis 3. More female dispersal associates with a later menopause

The dataset used to test the final hypothesis comprised of 876 women, who were primarily of duolocal descent (Figure 1c). As this dataset also included women who were premenopausal, it had a lower average age of 51.04 (*SD* = 12.85). As in the previous datasets, the majority of women had never smoked (96%) and had experienced some degree of financial insecurity (56%; Table S4).

Cox regression was used to measure the risk of entering menopause. There was no evidence to suggest women who leave their natal group, neither ancestrally nor currently, experience an earlier menopause, both prior to and after adjustment (Figure 2). In line with existing research, results did suggest that women who had never smoked (HR = 0.43, *p* < .01) and had a higher BMI (HR = 0.96, *p* < .01) experienced menopause at a later age (Table S5). As when comparing previous models (Table 2), based on the AIC the model that only included current residence pattern best fitted the data, even though this variable itself did not significantly associate with ANM.

## 4 DISCUSSION

Under the model of intragenomic conflict, it is predicted that, in populations where there is a greater degree of female dispersal, there should be a shorter, less symptomatic perimenopause that terminates in a later menopause. However, in this sample from four ethnic groups in China, we found little support for this hypothesis.

Firstly, it should be noted that the assumptions of the model of interest may be fundamentally flawed. Úbeda et al. (2014) justify aspects of their model by stating that, if prolonged survival following the cessation of fertility is an adaptive aspect of the human life history, then it is unclear why it is not a “rapid and smooth transition from the reproductive to the nonreproductive phases of a woman's life” (Úbeda et al., 2014:165). However, this does not have any theoretical underpinning. Many biological transitions seen in both humans and nonhumans are difficult and messy, and there is no reason to assume that the menopausal transition would be any different. In fact, many turbulent life events are important for fitness and selection (e.g., the birthing process, the transition from childhood to adolescence). Furthermore, in light of the differences in the menopausal transition observed between cultures and the many behavioral factors that have been found to influence symptom severity (Avis, Stellato, et al., 2001; Melby, Lock, & Kaufert, 2005), it may be that the magnitude of symptoms experienced by women (particularly Western women) is evolutionarily new. While this by no means renders the hypothesis moot, such point should be considered throughout the subsequent discussion.

We found no evidence for a difference in menopause symptom duration between the different modes of social organization, whether this be ancestral or current. In addition, there was no evidence that the duration of menopause symptoms was associated with the age at which women reached menopause (*r* = −.08, *p* = .51). This could be for a number of reasons. As stated, our sample is not large enough to capture small effects. Additionally, it may be that women are unable to accurately recall when they first begun experiencing menopause symptoms, particularly if it was a long time ago. Evidence for recall inaccuracy can be seen in Table S3, in which older women reported a significantly shorter perimenopause. Firstly, this may be because older women are unsure of when they started experiencing symptoms, resulting in a recall bias, and secondly, it may be that women who start experiencing symptoms at an older age experience a shorter perimenopause. Nonetheless, nonretrospective longitudinal data are most likely required in order to gain an accurate representation of the time in which women spend perimenopausal. It should also be noted that it may be that the initial model is incorrect in regards to when women experience symptoms. As previously mentioned, the intragenomic conflict model only addresses menopause symptoms prior to the termination of fertility; whereas in actuality, many women's symptoms persist for many years following menopause (Avis et al., 2015). There is scope for future research into the influence of intragenomic conflict on menopause symptom duration, and also what effects menopause symptom duration in general as there is currently little literature on the subject. However, at present, the data tested here showed no evidence for a difference in symptom duration between groups with different residence patterns.

With respect to age of menopause, once again, no relationship was observed. As can be seen in Figure 1f and l, there are peaks in reported age of menopause at age 40 and 50, which could be an indicator that some women are unsure of exactly when they had their last period and are therefore just approximating the timing. Hence, it may be that the insignificant relationship is due to a lack of clarity within the data. Although, the results do suggest that women who have smoked and have a lower BMI enter menopause earlier, which is in line with previous research (Ayatollahi et al., 2005; Bjelland et al., 2018; Gold et al., 2001, 2000; McKnight et al., 2011; Nagel et al., 2005; Özdemir & Çöl, 2004; Parazzini, 2007; Sapre & Thakur, 2014; Tao et al., 2015; Wang et al., 2018), suggesting that the data are, to an extent, capturing significant behavioral and lifestyle influences on menopause timing even if women were only estimating when they experienced menopause. We also observe that women who have more children report an earlier ANM, and on average women who live away from their natal group have higher parity. Currently, there is conflicting data on the relationship between menopause timing and fertility, with some research indicating that nulliparity or low fertility associates with an earlier menopause (Mishra et al., 2017; Mozumdar & Agrawal, 2014), and other reporting a negative relationship between number of children and ANM (Ahuja, 2016).

Finally, the model predicts that more female dispersal would result in less severe symptoms during the perimenopause; however, results from our data found the opposite. Prior to adjustment, women who were ancestrally and currently patrilocal reported more somato‐vegetative symptoms, with the same being found after adding covariates to the model. However, following adjustment and model fitting, the model that included current, rather than ancestral, residence pattern fit the data better. This negates Úbeda et al. (2014)'s hypothesis, which argues that ancestral ecology is responsible not only for the evolution of the human menopause, but also that it drives conflict between the woman's maternally and paternally inherited genes.

First, addressing the opposite relationship observed between ancestral residence and symptom severity, this could be partially due to confounding variables. A greater proportion of ancestrally patrilocal women report to having ever smoked regularly, and smoking is commonly identified as being a strong predictor of menopause symptoms (Avis, Crawford, & Green, 2018). Furthermore, ancestrally patrilocal women also had slightly higher parity within this sample, and the number of children a woman has has been observed to associate with increased menopause symptoms, possibly due to increased stress (Barth Olofsson & Collins, 2000; Blumel et al., 2000; Chedraui et al., 2010; Chlebowski et al., 2018; Jaber, Khalifeh, Bunni, & Diriye, 2017).

In addition to these possible confounding effects, we propose that the association between female dispersal and symptom severity may be due to the social differences that occur as a result of different current residence patterns. Within the study sample, the majority of women who reside in their natal household are Mosuo, and while there is a occasionally a degree of conflict between households, they are known for being an extremely peaceful group (Mace et al., 2018; Wu, Ji, et al., 2015). Ethnographic reports state that harmony is the ideal state within the household and that fissions between genetic kin are seldom observed and consciously avoided (Shih, 2009; Shih & Jenike, 2002). Furthermore, Mosuo women are highly autonomous (Liu & Zuo, 2018), which likely decreases their stress load making them better able to manage any negative menopause symptoms (Ahmad & Zakaria, 2015). On the other hand, there are more reports of social conflict among the Han and the Yi (Link, 2016). While research is limited, there is evidence that having a higher stress load associates with worse menopause symptoms (Barth Olofsson & Collins, 2000), and there is a large body of research, which highlights the effect of stress and conflict on general pain perception and management, with more stressful environments exacerbating the individuals sensitivity to the pain (Ahmad & Zakaria, 2015).

Compared to duolocal households, patrilocal households have lower levels of relatedness, which may decrease the drive to cooperate with one another. Sexual conflict (Trivers, 1972) can lead to tension between husbands and wives or women and their patrilineal relatives over fertility and mating decisions, which are more pronounced in patrilocal than matrilocal groups (Leonetti et al., 2007). It has been observed that when women live patrilocally, there is an increased level of spousal conflict that is often exacerbated by in‐laws, which may be because her husband's kin have less of an evolutionary interest in her (Leonetti et al., 2007; Skinner, 1997). This is exemplified in studies demonstrating that women who live with their matrilineal kin have higher fertility than women who do not and that women who live with their mothers are less likely to get divorced than those who live patrilocally (Leonetti et al., 2007; Sear, Mace, & McGregor, 2003). Furthermore, intimate partner violence is more often experienced by women in patrilineal and patrilocal societies (Sedziafa, Tenkorang, & Owusu, 2016, 2018), and as a result, sexual conflict and household dissonance may increase the woman's stress load, thus worsening a woman's menopausal transition. The positive effect of a relaxed environment on menopause symptoms is highlighted by the fact mindfulness and meditation are now being recommended by practitioners as a natural method of treatment (Wong et al., 2018).

## 5 CONCLUSION

We found no support for the predictions made in the intragenomic conflict hypothesis, with current residence pattern better predicting variation in aspects of the menopausal transition than ancestral residence pattern and results converse to the models predicted being found for menopause symptom severity. We propose that this may in part be the result of sexual and other social conflict, rather than intragenomic conflict, with a more stressful environment exacerbating the perimenopausal experience. We acknowledge that the results presented here are based on a small amount of data that nonetheless suggests that ecology and social organization may be an important predictor of menopause symptoms but not timing, but in the direction predicted from sexual conflict rather than genomic conflict.

## CONFLICT OF INTEREST

None declared.

## AUTHOR CONTRIBUTION

RM conceived the project. YY collected the data. Both MA and YY contributed equally to the preparation of the manuscript, and all authors contributed substantially to revisions.

## Data Availability

Data associated with this paper are available in Dryad, https://doi.org/10.5061/dryad.27s8k0p.
