# Supplementary material for: Current ecology, not ancestral dispersal patterns, influences menopause symptom severity
Source: Ecol Evol. 2019 Nov 5;9(22):12503–14. doi: 10.1002/ece3.5705 (PMC6875564; doi:10.1002/ece3.5705)
Supplement: Supplementary file 1 [file ECE3-9-12503-s001.docx]

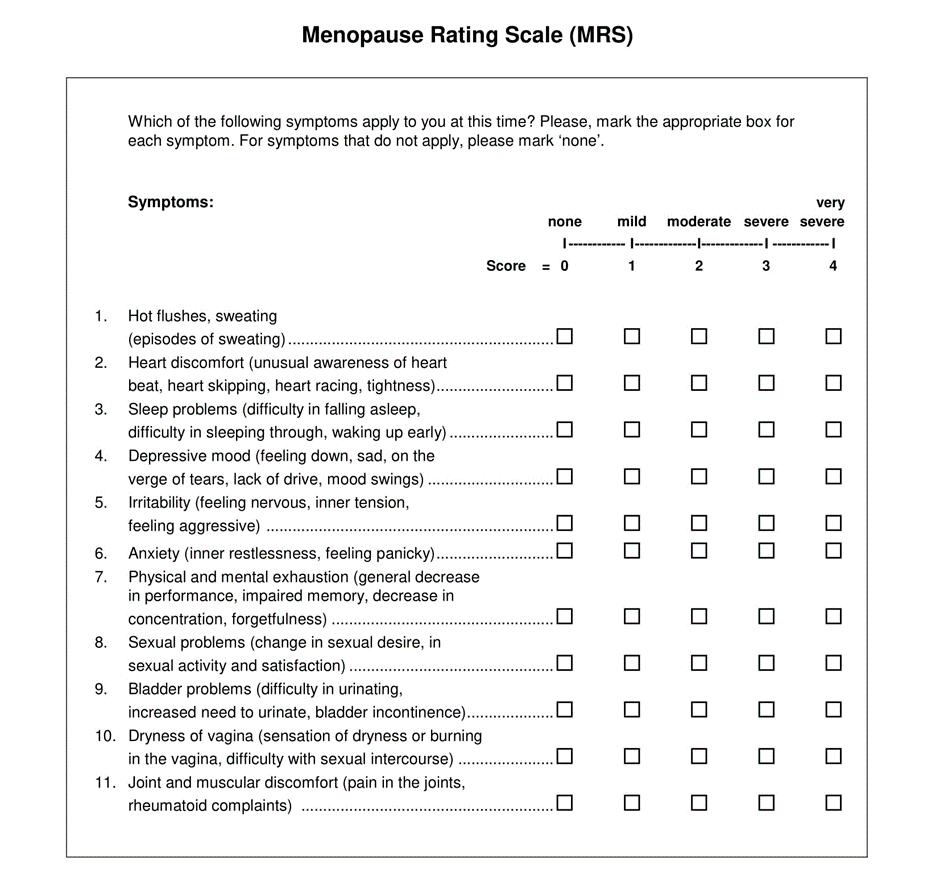


**Figure S1.** The Menopause Rating Scale used to measure menopause symptoms severity. Developed by Heinemann *et al.* (2003).

| **Table S1.** Full results from univariate and multivariate Poisson regression models where menopause somato-vegetative symptom severity is the outcome variable. Model 1 includes current residence pattern, ancestral residence pattern, age, menopause status, parity, financial security, smoking status, and body mass index (BMI); Model 2 includes all the aforementioned variables but with ancestral residence pattern removed; and Model 3 the same variables but with current residence pattern removed instead (n = 445). | | | | | | | | | |
| --- | --- | --- | --- | --- | --- | --- | --- | --- | --- |
|  | **Univariate** | | **H1 Model 1** | | **H1 Model 2** | | **H1 Model 3** | | |
|  | **β (S.E)** | **p** | **β (S.E)** | **p** | **β (S.E)** | **p** | **β (.E)** | **p** |  |
| Current residence pattern (ref.: Lives with natal group) |  |  |  |  |  |  |  |  |  |
| Lives away from natal group | 0.29 (0.04) | <0.01 | 0.23 (0.05) | <0.01 | 0.25 (0.04) | <0.01 | - | - |  |
| Ancestral residence pattern (ref.: Duolocal) |  |  |  |  |  |  |  |  |  |
| Mixed | 0.20 (0.10) | 0.03 | 0.18 (0.1) | 0.06 | - | - | 0.17 (0.1) | 0.08 |  |
| Patrilocal | 0.22 (0.05) | <0.01 | 0.05 (0.06) | 0.35 | - | - | 0.19 (0.05) | <0.01 |  |
| Age | -0.01 (0.00) | <0.01 | -0.02 (0.00) | <0.01 | -0.02 (0.00) | <0.01 | -0.02 (0.00) | <0.01 |  |
| Menopause status (ref.: Peri) |  |  |  |  |  |  |  |  |  |
| Post-menopausal | -0.27 (0.06) | <0.01 | -0.09 (0.07) | 0.22 | -0.08 (0.07) | 0.27 | -0.11 (0.07) | 0.14 |  |
| Parity | 0.04 (0.01) | <0.01 | 0.08 (0.01) | <0.01 | 0.08 (0.01) | <0.01 | 0.08 (0.01) | <0.01 |  |
| Experienced financial difficulty in past year (ref.: Yes) |  |  |  |  |  |  |  |  |  |
| No | -0.21 (0.05) | <0.01 | -0.20 (0.05) | <0.01 | -0.21 (0.05) | <0.01 | -0.02 (0.05) | <0.01 |  |
| Ever smoked (ref.: Yes) |  |  |  |  |  |  |  |  |  |
| No | 0.05 (0.09) | 0.55 | 0.18 (0.09) | 0.06 | 0.18 (0.09) | 0.05 | 0.17 (0.09) | 0.07 |  |
| BMI | 0.00 (0.00) | 0.43 | 0.00 (0.01) | 0.64 | 0.00 (0.01) | 0.51 | 0.00 (0.00) | 0.73 |  |
| *H1 = Hypothesis 1; S.E. = Standard error* | | | | | | | | |  |

| **Table S2.** Descriptive statistics of dataset used to test whether ancestral patrilocality associates with menopause symptom duration. | | | | | | | | |
| --- | --- | --- | --- | --- | --- | --- | --- | --- |
|  | **Total** | | **Ancestral residence pattern** | | | | | |
|  |  |  | **Duolocal (n = 41)** | | **Mixed (n = 3)** | | **Patrilocal (n = 39)** | |
|  | **n (%)** | **Mean (SD)** | **n (%)** | **Mean (SD)** | **n (%)** | **Mean (SD)** | **n (%)** | **Mean (SD)** |
| Symptom duration (years) |  | 1.75 (2.32) |  | 1.80 (2.42) |  | 1.17 (1.15) |  | 1.74 (2.33) |
| Age |  | 60.24 (7.17) |  | 58.98 (6.65) |  | 69.67 (5.51) |  | 60.85 (7.33) |
| Menopause status |  |  |  |  |  |  |  |  |
| Peri-menopausal | 13 (15.7) |  | 10 (24.4) |  | 0 (0.0) |  | 3 (7.7) |  |
| Post-menopausal | 70 (84.3) |  | 31 (75.6) |  | 3 (100.0) |  | 36 (92.3) |  |
| Current residence pattern |  |  |  |  |  |  |  |  |
| Lives with natal group | 28 (33.7) |  | 26 (63.4) |  | 2 (66.7) |  | 0 (0.0) |  |
| Lives away from natal group | 55 (66.3) |  | 15 (36.6) |  | 1 (33.3) |  | 39 (100.0) |  |
| Ancestral residence pattern |  |  |  |  |  |  |  |  |
| Duolocal | 41 (49.4) |  | 41 (100.0) |  | 0 (0.0) |  | 0 (0.0) |  |
| Mixed | 3 (3.6) |  | 0 (0.0) |  | 3 (100.0) |  | 0 (0.0) |  |
| Patrilocal | 39 (47.0) |  | 0 (0.0) |  | 0 (0.0) |  | 39 (100.0) |  |
| Parity |  | 3.99 (1.82) |  | 3.46 (1.53) |  | 6.67 (3.21) |  | 4.33 (1.77) |
| Experienced financial difficulty in past year |  |  |  |  |  |  |  |  |
| Yes | 57 (68.7) |  | 26 (63.4) |  | 3 (100.0) |  | 28 (71.8) |  |
| No | 26 (31.3) |  | 15 (36.6) |  | 0 (0.0) |  | 11 (28.2) |  |
| Ever smoked |  |  |  |  |  |  |  |  |
| Yes | 14 (16.9) |  | 3 (7.3) |  | 0 (0.0) |  | 11 (28.2) |  |
| No | 69 (83.1) |  | 38 (92.7) |  | 3 (100.0) |  | 28 (71.8) |  |
| BMI |  | 21.93 (3.20) |  | 22.93 (2.77) |  | 19.07 (3.09) |  | 21.10 (3.33) |
| *SD = Standard deviation; BMI = Body mass index* |  |  |  |  |  |  |  |  |

| **Table S3.** Full results from univariate and multivariate Cox regression models where menopause symptom duration is the outcome variable. Model 1 includes current residence pattern, ancestral residence pattern, age, menopause status, parity, financial security, smoking status, and body mass index (BMI); Model 2 includes all the aforementioned variables but with ancestral residence pattern removed; and Model 3 the same variables but with current residence pattern removed instead (n = 83). | | | | | | | | | | |
| --- | --- | --- | --- | --- | --- | --- | --- | --- | --- | --- |
|  | **Univariate** | | **Model 1** | | **Model 2** | | | **Model 3** | | |
|  | **HR (95% CI)** | **p** | **HR (95% CI)** | **p** | **HR (95% CI)** | **p** | **HR (95% CI)** | | **p** |  |
| Age | 1.06 (1.02, 1.09) | <0.01 | 1.06 (1.02, 1.10) | <0.01 | 1.06 (1.02, 1.10) | <0.01 | 1.06 (1.02, 1.10) | | <0.01 |  |
| Current residence pattern (ref.: Lives with natal group) |  |  |  |  |  |  |  | |  |  |
| Lives away from natal group | 1.09 (0.66, 1.79) | 0.75 | 0.98 (0.45, 2.11) | 0.95 | 1.06 (0.60, 1.86) | 0.85 | - | | - |  |
| Ancestral residence pattern (ref.: Duolocal) |  |  |  |  |  |  |  | |  |  |
| Mixed | 1.81 (0.55, 5.93) | 0.33 | 1.93 (0.44, 8.51) | 0.39 | - | - | 1.93 (0.44, 8.52) | | 0.39 |  |
| Patrilocal | 1.30 (0.80, 2.10) | 0.30 | 1.24 (0.59, 2.63) | 0.57 | - | - | 1.22 (0.69, 2.18) | | 0.50 |  |
| Parity | 1.05 (0.92, 1.19) | 0.47 | 0.89 (0.75, 1.06) | 0.20 | 0.92 (0.79, 1.08) | 0.32 | 0.89 (0.75, 1.06) | | 0.20 |  |
| Experienced financial difficulty in past year (ref.: Yes) |  |  |  |  |  |  |  | |  |  |
| No | 1.07 (0.64, 1.79) | 0.79 | 1.26 (0.71, 2.21) | 0.43 | 1.18 (0.68, 2.04) | 0.55 | 1.26 (0.72, 2.20) | | 0.42 |  |
| Ever smoked (ref.: Yes) |  |  |  |  |  |  |  | |  |  |
| No | 0.55 (0.31, 1.00) | 0.05 | 0.50 (0.25, 1.00) | 0.05 | 0.54 (0.27, 1.06) | 0.08 | 0.50 (0.25, 1.00) | | 0.05 |  |
| BMI | 0.98 (0.91, 1.06) | 0.57 | 1.02 (0.94, 1.12) | 0.60 | 1.01 (0.93, 1.10) | 0.79 | 1.02 (0.94, 1.12) | | 0.59 |  |
| *H2 = Hypothesis 2; CI = Confidence interval* |  |  |  |  |  |  |  | |  |  |

| **Table S4.** Descriptive statistics of dataset used to test whether patrilocality associates with age of menopause. | | | | | | | | | |
| --- | --- | --- | --- | --- | --- | --- | --- | --- | --- |
|  | **Total** | | **Ancestral residence pattern** | | | | | | |
|  |  |  | **Duolocal (n = 651)** | | | **Mixed (n = 34)** | | **Patrilocal (n = 191)** | |
|  | **n (%)** | **Mean (SD)** | **n (%)** | **Mean (SD)** | **n (%)** | | **Mean (SD)** | **n (%)** | **Mean (SD)** |
| Age |  | 51.04 (12.85) |  | 49.93 (12.84) |  | | 54.76 (14.35) |  | 54.17 (12.03) |
| Age of menopause |  |  |  | 48.39 (4.68) |  | | 49.38 (6.02) |  | 48.87 (3.79) |
| Menopause status |  |  |  |  |  | |  |  |  |
| Pre-menopausal | 446 (50.9) |  | 355 (54.5) |  | 13 (38.2) | |  | 78 (40.8) |  |
| Peri-menopausal | 46 (5.3) |  | 36 (5.5) |  | 0 (0.0) | |  | 10 (5.2) |  |
| Post-menopausal | 382 (43.6) |  | 259 (39.8) |  | 21 (61.8) | |  | 102 (53.4) |  |
| Don't know | 2 (0.2) |  | 1 (0.2) |  | 0 (0.0) | |  | 1 (0.5) |  |
| Current residence pattern |  |  |  |  |  | |  |  |  |
| Lives with natal group | 445 (50.8) |  | 414 (63.6) |  | 20 (58.8) | |  | 11 (5.8) |  |
| Lives away from natal group | 431 (49.2) |  | 237 (36.4) |  | 14 (41.2) | |  | 180 (94.2) |  |
| Ancestral residence pattern |  |  |  |  |  | |  |  |  |
| Duolocal | 651 (74.3) |  | 651 (100.0) |  | 0 (0.0) | |  | 0 (0.0) |  |
| Mixed | 34 (3.9) |  | 0 (0.0) |  | 34 (100.0) | |  | 0 (0.0) |  |
| Patrilocal | 191 (21.8) |  | 0 (0.0) |  | 0 (0.0) | |  | 191 (100.0) |  |
| Parity |  | 3.24 (1.73) |  | 3.05 (1.61) |  | | 3.94 (2.23) |  | 3.75 (1.91) |
| Experienced financial difficulty in past year |  |  |  |  |  | |  |  |  |
| Yes | 486 (55.5) |  | 337 (51.8) |  | 20 (58.8) | |  | 129 (67.5) |  |
| No | 390 (44.5) |  | 314 (48.2) |  | 14 (41.2) | |  | 62 (32.5) |  |
| Ever smoked |  |  |  |  |  | |  |  |  |
| Yes | 32 (3.7) |  | 12 (1.8) |  | 0 (0.0) | |  | 20 (10.5) |  |
| No | 844 (96.3) |  | 639 (98.2) |  | 34 (100.0) | |  | 171 (89.5) |  |
| BMI |  | 23.10 (4.58) |  | 23.29 (4.89) |  | | 22.43 (3.19) |  | 22.60 (3.56) |
| *SD = Standard deviation; BMI = Body mass index* | | | | | | | | | |

| **Table S5.** Full results from univariate and multivariate Cox regression models when age of menopause is the outcome variable. Model 1 includes current residence pattern, ancestral residence pattern, age, menopause status, parity, financial security, smoking status, and body mass index (BMI); Model 2 includes all the aforementioned variables but with ancestral residence pattern removed; and Model 3 the same variables but with current residence pattern removed instead (n = 876). | | | | | | | | | | |  |  |
| --- | --- | --- | --- | --- | --- | --- | --- | --- | --- | --- | --- | --- |
|  | **Univariate** | | **H3 Model 1** | | **H3 Model 2** | | | **H3 Model 3** | | | |  |
|  | **HR (95% CI)** | **p** | **HR (95% CI)** | **p** | | **HR (95% CI)** | **p** | | **HR (95% CI)** | **p** | | |
| Current residence pattern (ref.: Lives with natal group) |  |  |  |  | |  |  | |  |  | | |
| Lives away from natal group | 1.01 (0.82, 1.23) | 0.95 | 0.92 (0.73, 1.18) | 0.53 | | 0.94 (0.77, 1.15) | 0.56 | | - | - | | |
| Ancestral residence pattern (ref.: Duolocal) |  |  |  |  | |  |  | |  |  | | |
| Mixed | 1.08 (0.69, 1.69) | 0.73 | 1.03 (0.66, 1.62) | 0.89 | | - | - | | 1.03 (0.66, 1.61) | 0.90 | | |
| Patrilocal | 1.16 (0.92, 1.46) | 0.21 | 1.04 (0.78, 1.39) | 0.79 | | - | - | | 0.99 (0.78, 1.26) | 0.93 | | |
| Parity | 1.09 (1.04, 1.14) | <0.01 | 1.07 (1.02, 1.12) | 0.01 | | 1.07 (1.02, 1.12) | 0.01 | | 1.07 (1.02, 1.12) | 0.01 | | |
| Experienced financial difficulty in past year (ref.: Yes) |  |  |  |  | |  |  | |  |  | | |
| No | 0.98 (0.80, 1.20) | 0.86 | 1.00 (0.82, 1.23) | 0.97 | | 1.00 (0.82, 1.23) | 0.99 | | 1.00 (0.82, 1.23) | 0.99 | | |
| Ever smoked (ref.: Yes) |  |  |  |  | |  |  | |  |  | | |
| No | 0.43 (0.29, 0.63) | <0.01 | 0.51 (0.34, 0.76) | <0.01 | | 0.50 (0.34, 0.74) | <0.01 | | 0.50 (0.34, 0.75) | <0.01 | | |
| BMI | 0.96 (0.94, 0.99) | <0.01 | 0.97 (0.94, 0.99) | 0.01 | | 0.97 (0.94, 0.99) | 0.01 | | 0.97 (0.94, 0.99) | 0.01 | | |
| *H3 = Hypothesis 3; CI = Confidence interval* | | | | | | | | | | |  |  |
